# Therapeutic communication in nursing students: A cross-sectional study of personal, educational, and contextual factors

**DOI:** 10.1371/journal.pone.0345109

**Published:** 2026-03-17

**Authors:** Jaime Carballedo-Pulido, Mariona Farrés-Tarafa, Juan Roldán-Merino, Bárbara Hurtado-Pardos, Marta Berenguer-Poblet, Manuel Tomás-Jiménez, Carla Otero-Arús, Marta Domínguez-del-Campo, Ivette Fernández-Gibert, Susana Santos-Ruiz

**Affiliations:** 1 Campus Docent Sant Joan de Deu, Universitat Vic-Universitat Central de Catalunya (UVIC-UCC), Barcelona, Spain; 2 Department of Fundamental and Clinical Care Nursing, Hospitalet del Llobregat, Universitat de Barcelona, Campus de Bellvitge, Barcelona, Spain; 3 Mental Health, Psychosocial and Complex Nursing Care Research Group-2021 SGR 01083, Barcelona, Spain; 4 Nursing Department, Campus Terres de l’Ebre, Universitat Rovira i Virgili, Tortosa, Spain; 5 Research Group on Advanced Nursing (CARING)-161, Universitat Rovira I Virgili, Tarragona, Spain; 6 Patient Safety Research Group, Parc Sanitari Sant Joan de Déu, Sant Boi de Llobregat, Spain; 7 Parc Sanitari Sant Joan de Déu, Sant Boi de Llobregat, Spain; 8 Parc Taulí Hospital Universitari. Institut d’Investigació i Innovació Parc Taulí (I3PT-CERCA), Universitat Autònoma de Barcelona. Sabadell, Spain; School of Nursing Sao Joao de Deus, Evora University, PORTUGAL

## Abstract

**Background:**

Therapeutic communication is a key competency in nursing education and a central component of person-centred care. However, evidence regarding its association with personal, educational, and contextual factors among nursing students remains heterogeneous.

**Objective:**

To describe therapeutic communication scores in nursing students and to examine their association with personal, educational, and contextual variables using the Therapeutic Communication Scale in Nursing Students.

**Methods:**

A cross-sectional study was conducted among second-, third-, and fourth-year undergraduate nursing students at a Spanish university. Participants completed a questionnaire including sociodemographic and academic variables, self-perceived communication ability, factors related to stress and the clinical practice context, and the Therapeutic Communication Scale in Nursing Students, which provides a total score and two dimensions: Relation Building and Problem Solving. Descriptive statistics were calculated, and bivariate analyses were performed using non-parametric tests.

**Results:**

A total of 450 students participated. Mean scores indicated a generally high level of therapeutic communication. Female students obtained significantly higher scores than male students in both dimensions and in the total scale score. No significant differences were observed according to academic shift, employment status, volunteering experience, or academic stress level. Higher self-perceived communication ability and greater motivation towards nursing studies were associated with higher therapeutic communication scores. In addition, students who reported frequent use of communicative behaviours such as active listening, verbal empathy, and open-ended questions showed significantly higher scores. Perceived support from clinical tutors and clinical accompaniment was not associated with communication scores, whereas a favourable clinical climate was associated with slightly higher total scores.

**Conclusions:**

Therapeutic communication in nursing students appears to be more strongly associated with self-perceived competence, academic motivation, and specific communicative behaviours than with sociodemographic characteristics or stress levels. These findings highlight the importance of strengthening training in concrete communication skills during undergraduate nursing education.

## Introducción

Clinical communication is an essential competency in health professions and a central component of person-centred care. Effective communication plays a key role in healthcare. It helps patients understand clinical information, supports their participation in decisions, and strengthens the therapeutic relationship, which directly influences the quality and safety of care [1,2]. In nursing practice, therapeutic communication goes beyond simply sharing information. It involves building a respectful and empathetic connection with patients, encouraging their active involvement, and creating a space where they can express concerns, emotions, and health-related needs [3,4]. Research has shown that strong therapeutic communication fosters trust, improves the patient experience, and is linked to better care outcomes, especially in long-term care, mental health services, and chronic disease management [5,6].

Although clinical communication is often used as an umbrella term encompassing both relational behaviours and the structured exchange of clinical information [7], therapeutic communication represents a distinct relational component [8]. It involves behaviours such as active listening, verbal empathy, and the exploration of patient concerns, which aim to build trust and support person‑centred care. This contrasts with information‑transfer communication, which focuses on accuracy, structure, and patient‑safety processes such as handovers or clinical briefings [9]. The present study focuses specifically on therapeutic communication as a relational skill set relevant to nursing practice.

In undergraduate health professions education, communication is conceptualised as a set of observable and teachable skills. Among the most widely studied behaviours are active listening, the use of open-ended questions, verbal empathy, clarification of information, and the ability to structure the clinical interview [1,10]. These skills fulfil complementary functions because they operate across two distinct yet interrelated domains. On the one hand, they strengthen the relational dimension of care by fostering a therapeutic relationship grounded in trust and mutual respect. On the other hand, they support the clinical and instrumental dimension by enabling more accurate information gathering, identifying patient-relevant concerns, and facilitating shared decision-making. Conceptual literature on therapeutic communication identifies information exchange, engagement, mutual respect, and the management of patient-relevant concerns as core attributes, supporting the examination of therapeutic communication through differentiated but connected dimensions [2].

Empirical evidence among healthcare students and professionals shows heterogeneous findings regarding the level of communication skills and their associated factors.

Structured clinical assessments have reported differences by sex, with female students often showing stronger performance in areas such as empathy, communication organisation, and both verbal and non-verbal expression [11]. Comparable patterns have been described in dentistry, where women, as well as professionals with greater age or clinical experience, tend to obtain higher therapeutic communication scores [12]. However, findings are not consistent across all health disciplines. Several studies in student populations have found no clear relationship between communication skills and variables such as age, sex, or academic year, suggesting that training context and educational approaches may have a stronger influence than demographic factors alone [13].

Within nursing education, previous care experience or employment in healthcare has been considered a possible contributor to the development of relational competence. Yet, evidence remains mixed. Some research has identified early differences in communication self-efficacy linked to prior experience, although these gaps often narrow as students progress through their studies [14]. Similarly, mixed-methods work has not shown lasting quantitative advantages in compassionate values or behaviours, even when participants report that earlier experience felt helpful [15]. These results question whether clinical exposure by itself is enough to strengthen communication skills and highlight the need for structured teaching and intentional practice [16,17].

Beyond demographic and experiential factors, psychological and educational variables have become central in therapeutic communication research. Self-efficacy is widely used to capture learners’ confidence in their communication abilities and appears responsive to training interventions [18,19]. Systematic reviews suggest that communication programmes can improve both confidence and observed performance, particularly when they rely on experiential learning, simulation-based activities, and regular feedback [20,21]. In addition, associations have been reported among nursing students between academic motivation and the application of therapeutic communication, suggesting that learners’ readiness and level of engagement may influence the extent to which these skills are translated into clinical practice [22].

Academic stress represents another relevant factor in nursing education. Several studies have documented high levels of stress among nursing students and its associations with variables such as resilience, emotional responses, academic self-efficacy, and self-directed learning [23–26]. However, there is limited evidence specifically examining the relationship between academic stress and therapeutic communication skills assessed using structured measurement instruments, representing a relevant gap in the existing literature.

Beyond individual factors, the clinical practice context may also influence communication. Among healthcare professionals, an association has been described between the practice environment and the quality of the therapeutic relationship, as well as between organisational climate and positive relational behaviours [6]. Qualitative studies conducted in other clinical settings indicate that organisational and environmental factors shape the perception of a patient-centred therapeutic relationship [27]. Among students, variables such as perceived workplace climate, tutor support, and clinical supervision may facilitate or hinder the expression of communication skills, although the available empirical evidence remains limited.

An additional challenge in this field concerns the measurement of therapeutic communication. Multiple self-report instruments are available, with heterogeneous theoretical foundations and variable psychometric properties. Systematic reviews have highlighted substantial variability in the dimensions assessed, as well as in the validity and reliability of existing scales [4]. The Therapeutic Communication Scale in Nursing Students [28] offers a structured approach to measuring therapeutic communication in undergraduate nursing students. It provides an overall score and includes two clinically meaningful components: Relation Building, which focuses on interpersonal connection, and Problem Solving, which reflects communication aimed at addressing patient needs. This two-domain model makes it possible to explore how communication skills relate to a range of individual, educational, behavioural, and environmental factors during training.

In university nursing programmes, understanding students’ communication levels and the factors linked to stronger performance can support the development of more effective teaching and support strategies. Behaviours such as active listening and empathic verbal responses may be particularly relevant, as well as broader influences such as academic motivation, perceived stress, and the quality of the clinical placement setting. For this reason, the present study aimed to describe therapeutic communication scores among nursing students and to examine their associations with personal, educational, and contextual variables, using the scale and its two dimensions, Relation Building and Problem Solving.

## Methods

### Study design

We carried out an observational cross-sectional study to explore therapeutic communication among nursing students and its relationship with personal, educational, and contextual factors. Therapeutic communication was assessed using the Therapeutic Communication Scale in Nursing Students, which provides an overall score and two specific domains: Relation Building and Problem Solving. In all cases, higher scores reflected stronger therapeutic communication skills.

### Study setting and sample

The study was conducted at the Campus Docent Sant Joan de Déu, an affiliated centre of the Universitat de Vic–Universitat Central de Catalunya, among undergraduate nursing students enrolled in the second, third and fourth academic years during the 2025–2026 academic year. Data collection took place between 10 November and 15 December 2025. A non-probabilistic convenience sampling approach was used, inviting all students present in the classroom during the data collection period who met the inclusion criterion of having completed at least one period of clinical placement in a hospital or community setting. Students who were absent on the day of questionnaire administration or who had incomplete data on the main study variables were excluded.

Data were collected using a structured self-administered questionnaire composed of two sections. The first section gathered sociodemographic and academic information, as well as variables related to training background, self-perceived communication ability, communication behaviours, and characteristics of the clinical practice context. The second section included the *Therapeutic Communication Scale in Nursing Students* [28], which yields a total score and factor-specific scores.

### Sample size

The required sample size was estimated according to the main analytical aim of the study, which was to explore group differences and associations in therapeutic communication scores using non-parametric methods. With an alpha level of 0.05, a power of 80%, and an expected small-to-moderate effect (r around 0.30), we determined that a sample of about 350 students would be sufficient. In total, 450 nursing students were included, providing adequate precision to identify meaningful differences in both the overall scale score and its two dimensions.

### Variables and sources of information

Data were collected on a range of personal, academic, and educational characteristics. Sociodemographic information included age, sex, academic year, and enrolment schedule. We also recorded whether students were employed at the time of the study and, if so, whether their work was related to healthcare. Motivation for entering the nursing degree was assessed and classified as either a personal decision or influenced by external recommendations.

Several educational and perception-based variables were also examined. Students rated their own communication ability using four response options (very poor, poor, good, very good). For the analyses, these categories were combined into two broader groups (very poor/poor vs. good/very good). Additional measures included motivation towards nursing studies, perceived academic stress, and how important students considered therapeutic communication in clinical practice.

Self-reported communication behaviours were assessed, including active listening, paraphrasing, verbal empathy, and the use of open-ended questions. Responses were grouped into two categories (never/almost never vs. always/almost always). Clinical placement factors were also included, such as perceived support from clinical tutors, supervision by clinical nurses, and the overall workplace climate. These items were analysed by collapsing responses into dissatisfaction or satisfaction levels.

Therapeutic communication was measured with the Therapeutic Communication Scale in Nursing Students [28]. This 15-item instrument yields an overall score and two domain scores. The Relation Building dimension includes nine items focused on establishing the therapeutic relationship, while the Problem Solving dimension consists of six items assessing communication skills used to address patient needs during clinical encounters.

Items are rated on a four-point Likert scale from 1 (strongly disagree) to 4 (strongly agree). Total scores range from 15 to 60, and dimension scores are calculated by summing the relevant items. Higher scores indicate stronger therapeutic communication skills.

### Procedure

Questionnaire administration was organised in collaboration with the academic staff. Data collection sessions were scheduled across academic years and enrolment groups to ensure that all eligible students could participate. Before completing the survey, the research team provided standardised information about the study aims, the voluntary nature of participation, and the confidentiality of responses. Students were also informed that choosing not to participate would not affect their academic evaluation.

Data were gathered through a self-administered online questionnaire delivered via the REDCap platform. This system allowed secure data handling, ensured anonymity, and supported structured storage of the collected information. Students completed the questionnaire individually within the allocated time, without interference from the research team. Only questionnaires that were completed in a valid manner were included in the analysis.

### Data analysis

A descriptive analysis was performed for all study variables. Quantitative variables were summarised using means and standard deviations, as well as minimum and maximum values. Categorical variables were described using absolute frequencies and percentages.

As the scores for Factor 1 (*Relation Building*), Factor 2 (*Problem Solving*), and the total therapeutic communication score did not meet the assumption of normality according to the Shapiro–Wilk test (p < 0.001 for all), non-parametric methods were applied for the bivariate analyses.

Comparisons of therapeutic communication scores between two independent groups were conducted using the Mann–Whitney U test. Effect sizes were calculated using the rank-biserial correlation. A significance level of p < 0.05 was applied in all analyses.

All statistical analyses were performed using jamovi software (version 2.6), based on R.

### Ethical considerations

In addition to approval by the Research Ethics Committee of Campus Docent Sant Joan de Déu PR2 25, informed consent was obtained from all participants prior to data collection. Students received oral information about the study during class and were provided with a detailed written information sheet through the REDCap platform. The online form included the informed consent document, which participants were required to read and electronically sign before accessing the questionnaire. A downloadable copy of the signed consent form was made available to each participant. Participation was entirely voluntary. Students were informed that they could decline to participate or withdraw at any time without academic consequences. Only questionnaires submitted after signed informed consent were included in the analysis.

## Results

### Descriptive analysis of the sample

The sociodemographic and academic characteristics of the participants are shown in Table 1. The sample comprised 450 students, with a mean age of 22.7 years (SD = 4.4). Female students predominated (83.1%). Most participants were enrolled in the second (37.8%) and third academic years (39.3%), followed by the fourth year (22.9%). Regarding enrolment schedule, 65.3% attended morning classes and 34.7% afternoon classes. More than half of the students reported being employed at the time of the study (61.6%), and approximately half of these worked in hospital or residential care settings (50.9%). In addition, 32.7% reported previous volunteer experience in hospital or residential environments. The majority of participants indicated that nursing was their personal career choice (94.7%).

**Table 1:** Sociodemographic and academic characteristics of the participants (n = 450).

	n	%
**Age (years), mean (SD)**	22,7 (4,4)	
**Sex**		
Female	374	83,1
Male	76	16,9
**Academic year**		
Second year	170	37,8
Third year	177	39,3
Fourth year	103	22,9
**Enrolment schedule**		
Morning	294	65,3
Afternoon	156	34,7
**Currently employed**		
Yes	277	61,6
No	173	38,4
**Employment in hospital or residential care**		
Yes	141	50,9
No	136	49,1
**Volunteer experience in hospital or residential settings**		
Yes	147	32,7
No	303	67,3
**Motivation for choosing nursing degree**		
Personal choice	426	94,7
External recommendation	24	5,3

### Descriptive analysis of educational characteristics and clinical practice context

Educational characteristics, perceived communication competence, and the clinical practice context are summarised in Table 2. Most students reported a good or very good level of self-perceived communication ability (87.6%). Regarding motivation towards their nursing education, 91.1% of participants reported being motivated or highly motivated. In terms of perceived academic stress or workload, 62.2% of students indicated high or very high levels. The perceived importance of therapeutic communication was predominantly rated as high or very high (96.9%).

**Table 2:** Educational characteristics, perceived communication competence, and clinical practice context (n = 450).

Variable	n	%
**Self-perception and education**		
**Self-perceived communication ability**		
Very poor or poor	56	12,4
Good or very good	394	87,6
**Motivation towards nursing education**		
Very unmotivated or unmotivated	40	8,9
Motivated or highly motivated	410	91,1
**Perceived academic stress or workload**		
Very low or low	170	37,8
High or very high	280	62,2
**Perceived importance of therapeutic communication**		
Very low or low	14	3,1
High or very high	436	96,9
**Communication behaviours**		
**Active listening**		
Never or almost never	9	2.0
Always or almost always	441	98.0
**Paraphrasing**		
Never or almost never	256	56,9
Always or almost always	194	43,1
**Verbal empathy**		
Never or almost never	31	6,9
Always or almost always	419	93,1
**Use of open-ended questions**		
Never or almost never	208	46,2
Always or almost always	242	53,8
**Clinical practice context**		
**Perceived support from clinical tutors**		
Dissatisfied or very dissatisfied	162	36,0
Satisfied or very satisfied	288	64,0
**Perceived supervision by the clinical nurse**		
Dissatisfied or very dissatisfied	77	17,1
Satisfied or very satisfied	373	82,9
**Perceived workplace climate during last clinical placement**		
Dissatisfied or very dissatisfied	99	22,0
Satisfied or very satisfied	351	78,0

Regarding self-reported communication behaviours, most participants indicated engaging in active listening always or almost always (98.0%) and demonstrating verbal empathy with the same frequency (93.1%). The use of open-ended questions was reported as frequent by 53.8% of students, whereas paraphrasing was used always or almost always by 43.1%. Concerning the clinical practice context, 64.0% of participants reported being satisfied or very satisfied with the support provided by clinical tutors, 82.9% with supervision by the clinical nurse, and 78.0% with the workplace climate of the healthcare setting.

### Descriptive scores of therapeutic communication

Descriptive scores for therapeutic communication are presented in Table 3. The mean total score of the Therapeutic Communication Scale in Nursing Students was 51.0 points (SD = 6.4). Regarding the scale dimensions, the mean score for Factor 1 (Relation Building) was 30.5 points (SD = 3.9), while the mean score for Factor 2 (Problem Solving) was 19.0 points (SD = 3.0). In all cases, higher scores indicate a higher level of therapeutic communication skills.

**Table 3:** Descriptive statistics of the Therapeutic Communication Scale in Nursing Students and its dimensions.

	Min.	Max.	Mean	SD
Factor 1. Relation Building	9,0	36,0	30,5	3,9
Factor 2. Problem Solving	6,0	24,0	19,0	3,0
Total scale	15,0	60,0	51,0	6,4

**Note:** Higher scores indicate a higher level of therapeutic communication skills.

### Bivariate analysis of therapeutic communication scores

The associations between personal, educational, and contextual variables and scores on the Therapeutic Communication Scale in Nursing Students are presented in Table 4. As the scale scores did not meet the assumption of normality, Mann–Whitney U tests were used for comparisons between independent groups.

**Table 4:** Association between personal, educational, and contextual variables and Therapeutic Communication Scale in Nursing Students scores (n = 450).

Variable	Group	n	Factor 1 Mean (SD)	Factor 2 Mean (SD)	Total scale Mean (SD)	p	r
Sex	Female	374	30,9 (3,74)	19,3 (2,97)	50,1 (6,16)	<0,001	0,26
	Male	76	29,1 (4,64)	18,3 (3,14)	47,4 (7,16)		
Self-perceived communication skills	Poor or very poor	56	29,1 (3,87)	18,5 (3,24)	47,6 (6,39)	<0,001	0,32
	Good or very good	394	30,8 (3,93)	19,2 (2,98)	50,0 (6,36)		
Motivation towards training	Unmotivated	40	28,7 (4,12)	17,6 (3,37)	46,3 (6,86)	<0,001	0,41
	Motivated	410	30,8 (3,89)	19,2(2,95)	50,0 (6,28)		
Active listening	Never or almost never	9	28,6 (2,13)	17,1 (3,02)	45,7 (3,64)	<0,004	0,56
	Always or almost always	441	30,6 (3,97)	19,1 (3,01)	49,7(6,43)		
Verbal empathy	Never or almost never	31	28,4 (3,87)	17,6 (2,87)	46,0 (5,47)	0,002	0,47
	Always or almost always	419	30,7 (3,92)	19,2 (3,00)	49,9 (6,40)		
Use of open-ended questions	Never or almost never	208	30,0 (3,51)	18,7 (2,74)	48,7 (5,57)	<0,001	0,25
	Always or almost always	242	31,0 (4,25)	19,5 (3,20)	50,5 (6,96)		
Work environment	Dissatisfied or very dissatisfied	99	30,0 (4,65)	18,6 (3,30)	48,6 (7,33)	0,048	0,13
	Satisfied or very satisfied	351	30,7 (3,73)	19,2 (2,92)	50,0 (6,10)		

**Notes: p** values obtained using the Mann–Whitney U test. **r** corresponds to the rank-biserial correlation (effect size). Higher scores indicate higher levels of therapeutic communication skills.

Statistically significant differences in therapeutic communication scores were observed according to sex. Female students obtained higher scores than male students for Factor 1 (Relation Building) (30.9 ± 3.74 vs. 29.1 ± 4.64), Factor 2 (Problem Solving) (19.3 ± 2.97 vs. 18.3 ± 3.14), and the total scale score (50.1 ± 6.16 vs. 47.4 ± 7.16), with small to moderate effect sizes (r = 0.26).

No statistically significant differences in therapeutic communication scores were found according to enrolment schedule, current employment status, volunteer experience, or motivation for choosing nursing as a career (p > 0.05).

Regarding educational variables, students reporting better self-perceived communication ability showed significantly higher scores in both factors and in the total scale score. Specifically, students with good or very good self-perceived communication ability obtained a higher mean total score (50.0 ± 6.36) compared with those reporting poor or very poor ability (47.6 ± 6.39; p < 0.001; r = 0.32). Similarly, students who reported being motivated towards their nursing education achieved higher total scale scores than those who were unmotivated (50.0 ± 6.28 vs. 46.3 ± 6.86), with a moderate effect size (r = 0.41).

No statistically significant differences were observed in Factor 1, Factor 2, or total therapeutic communication scores according to the level of perceived academic stress (p > 0.05 in all cases).

With respect to communication behaviours, students who reported engaging in active listening always or almost always obtained higher total scale scores (49.7 ± 6.43) than those who reported doing so never or almost never (45.7 ± 3.64), with a large effect size (r = 0.56). Similarly, frequent use of verbal empathy was associated with higher total scores (49.9 ± 6.40 vs. 46.0 ± 5.47; p = 0.002; r = 0.47). Frequent use of open-ended questions was also associated with higher therapeutic communication scores (50.5 ± 6.96 vs. 48.7 ± 5.57; p < 0.001; r = 0.25).

Finally, no statistically significant differences in therapeutic communication scores were observed according to perceived support from clinical tutors or clinical supervision (p > 0.05). However, a favourable perceived workplace climate was associated with higher total scale scores (50.0 ± 6.10 vs. 48.6 ± 7.33), although the effect size was small (r = 0.13).

## Discussion

This study examined therapeutic communication scores among nursing students and their associations with personal, educational, and contextual variables using the Therapeutic Communication Scale in Nursing Students. Overall, the findings indicate that therapeutic communication is more strongly associated with educational and behavioral factors than with sociodemographic or employment-related characteristics. This pattern suggests that the development of therapeutic communication during undergraduate nursing education may depend largely on modifiable and trainable factors rather than on stable personal attributes.

Regarding personal variables, significant differences were observed according to sex, with female students obtaining higher scores than male students on the total scale as well as on both dimensions, Relation Building and Problem Solving. However, the effect sizes were small to moderate, indicating that although the differences were consistent, their magnitude was limited.

Our results align with earlier studies in both healthcare students and practising professionals, where women have often reported or demonstrated stronger communication skills, particularly in areas linked to empathy, verbal expression, and interpersonal engagement [11,12,29]. However, the existing evidence suggests that these differences are more likely shaped by sociocultural and educational factors, such as gendered socialisation and learning opportunities, rather than by biological or inherent traits. For this reason, the differences observed in our sample should be interpreted with caution and understood within their broader contextual and formative background.

In our study, therapeutic communication scores did not differ significantly by academic shift, employment status, previous volunteering, or the motivation for choosing nursing. This pattern suggests that simple exposure to healthcare environments, whether through work or volunteering, may not be sufficient to enhance communication skills on its own. Instead, these findings support the view that therapeutic communication develops more effectively through structured and intentional training approaches [13–15].

Educational factors showed more consistent relationships with therapeutic communication. Students who rated their own communication abilities more positively achieved higher scores on the total scale and across both dimensions, with small to moderate effect sizes [19,20]. This suggests a degree of agreement between students’ perceived competence and their performance on a validated measure. At the same time, the cross-sectional design prevents drawing conclusions about the direction of this relationship [4,30,31].

Higher academic motivation was also linked to stronger therapeutic communication scores. This is in line with previous research indicating that more motivated students tend to apply therapeutic communication strategies more consistently during clinical practice [21,22]. Overall, these findings underline the importance of academic engagement and self-efficacy in supporting the development of communication competencies in nursing education. Motivation and confidence in one’s own abilities may facilitate the active use and consolidation of therapeutic communication skills during training and clinical exposure [2,20,22].

Regarding perceived academic stress among nursing students, no statistically significant differences were observed in therapeutic communication scores. This finding suggests that, in this specific sample, academic stress was not directly associated with lower levels of communication skills. However, previous research has consistently shown that academic stress negatively affects other domains, including learning approaches, academic motivation, procrastination, and physical and psychological well-being [23–26,32]. Therefore, it cannot be ruled out that the impact of stress on therapeutic communication may emerge in other educational contexts, such as more complex clinical placements, or become evident only through longitudinal designs and studies with larger samples.

Self-reported communicative behaviours showed the strongest associations with therapeutic communication. Active listening, verbal empathy, and frequent use of open-ended questions were associated with significantly higher scores on the total scale and across both dimensions, with moderate and, in some cases, large effect sizes. These findings reinforce the relevance of these behaviours as core components of therapeutic communication and highlight the need to explicitly promote them within nursing education. This is consistent with previous evidence linking active listening, empathy, and open-ended questioning to stronger clinician–patient relationships, greater patient emotional expression, and improved health-related outcomes [1,5,10,33,34].

In contrast, paraphrasing showed a more limited association with therapeutic communication scores. This may suggest that paraphrasing is used less frequently by students or is more difficult to integrate during early clinical practice, despite evidence from other educational and clinical contexts indicating that its appropriate use enhances patients’ sense of being understood and increases perceived professional empathy [3,35,36]

Regarding the clinical placement context, no statistically significant associations were observed between therapeutic communication scores and students’ perceptions of tutor support or clinical supervision. This finding is consistent with previous studies suggesting that communication skills are often acquired in a transversal and weakly standardised manner, and that their development depends largely on individual factors and the hidden curriculum rather than on isolated or punctual instructional support [17,37].

In contrast, the perception of a favourable work climate was associated with slightly higher total scores on the therapeutic communication scale, although the effect size was small. This result aligns with evidence linking more supportive practice environments and better work organisation to higher-quality therapeutic relationships [6,27]. Overall, these findings suggest that the relational and organisational environment may facilitate the expression of communication skills. However, its influence appears to be secondary when compared with individual and behavioural variables, such as professional attitudes, deliberate practice, and structured training in communication skills [6,16,38,39].

Several limitations of this study should be considered. First, because of the cross-sectional design, it is not possible to determine causal relationships between the variables examined. Second, communication skills were assessed through self-reported measures, which may be influenced by social desirability and could result in an overestimation of students’ abilities. In addition, the use of a non-probabilistic sampling approach restricts the extent to which these findings can be generalised to other nursing student populations. Despite these limitations, the relatively large sample and the inclusion of students across different academic years add robustness to the study.

From an applied standpoint, our results indicate that therapeutic communication training should focus on developing concrete behaviours, such as active listening, verbal empathy, and the use of open-ended questions, rather than assuming that clinical exposure alone is sufficient. Longitudinal research is needed to better understand how these skills evolve during nursing education and to assess the impact of structured, targeted training interventions over time.

## Conclusions

This study describes therapeutic communication skills in nursing students and explores how these skills relate to personal, educational, and contextual factors using the Therapeutic Communication Scale in Nursing Students. Overall, participants reported relatively high levels of therapeutic communication, underscoring the importance of this competence within undergraduate nursing training.

Therapeutic communication was more closely linked to educational and behavioural variables than to sociodemographic or work-related characteristics. Female students achieved higher scores than male students, although the size of this difference was small to moderate. In contrast, no significant associations were found for academic shift, employment status, previous volunteering experience, or academic stress.

Students’ self-perceived communication ability and academic motivation were both associated with higher therapeutic communication scores, suggesting that confidence and engagement with learning may support the development of these competencies. In particular, specific behaviours such as active listening, verbal empathy, and the use of open-ended questions showed the strongest relationships with communication outcomes, highlighting their relevance in day-to-day clinical interactions.

Regarding the clinical practice context, perceptions of tutor support and clinical supervision were not significantly related to communication skills, whereas a more favourable work climate was associated with slightly higher overall communication scores. This suggests that the organisational and relational environment may facilitate the expression of communication skills, although its influence appears to be secondary compared with individual and behavioural factors.

Taken together, these findings underline the importance of reinforcing structured training in specific communicative behaviours within nursing education, beyond reliance on clinical exposure alone. Strengthening students’ motivation, self efficacy and deliberate practice of core communication skills may contribute to more effective therapeutic communication and improved quality of care.

## Supporting information

S1 Data The dataset is provided as supporting information (S1_Dataset). (XLSX)
